# Medial and lateral discoid menisci: a case report

**DOI:** 10.1186/1758-2555-2-21

**Published:** 2010-08-23

**Authors:** Sung-Jae Kim, Andri MT Lubis

**Affiliations:** 1 Department of Orthopaedic Surgery, Yonsei University College of Medicine, 250-Seongsanno, Seodaemun-gu, Seoul 120752, South Korea; 2 Division of Orthopaedic and Traumatology, Department of Surgery, Faculty of Medicine University of Indonesia, Salemba Raya 6, Jakarta 10430, Indonesia

## Abstract

Discoid menisci on both medial and lateral tibial plateau are very rare abnormalities. We report a 44-year-old woman with bilateral medial and lateral discoid menisci. She also had anomalous insertion of discoid medial meniscus to anterior cruciate ligament, and pathologic medial patellar plica on the right knee. Meniscectomies has been performed for her torn discoid menisci with satisfactory result on the latest follow-up.

## Background

In 1889, Young described a lateral discoid meniscus in a cadaver study [1], whereas medial discoid meniscus was reported at the first time by Cave and Staples in 1941 [2]. However, up to recent years medial discoid meniscus cases were still very rarely reported. The first case of both medial and lateral discoid menisci in the same knee was reported by Jeannopolous in 1950 [3]. Murdoch reported the first case of bilateral medial discoid menisci in 1956 [4,5] and afterwards bilateral medial discoid menisci are extremely rarely reported [5-14]. We report a case of bilateral medial and lateral discoid menisci. The patient did well postoperatively after both medial menisci were excised in two separate operations.

## Case report

**Figure 1:**
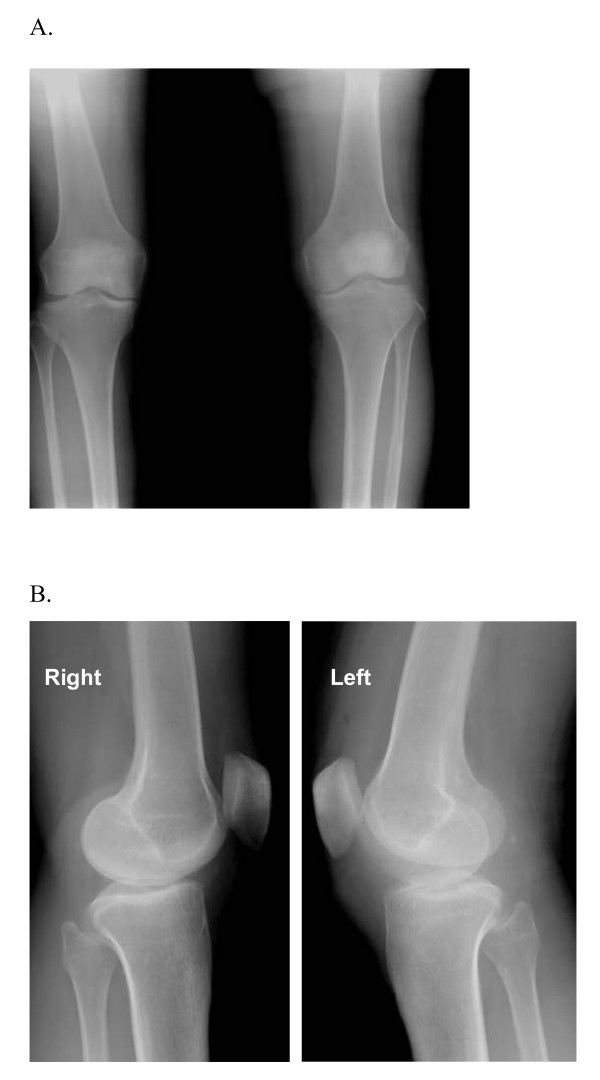
**Anteroposterior and lateral standing radiographs of the both knee**. (A) Anteroposterior and (B) lateral (right and left) radiographs showing bilateral widening of lateral joint spaces, and increased concavity and subchondral sclerotic of the medial tibial plateau.

**Figure 2:**
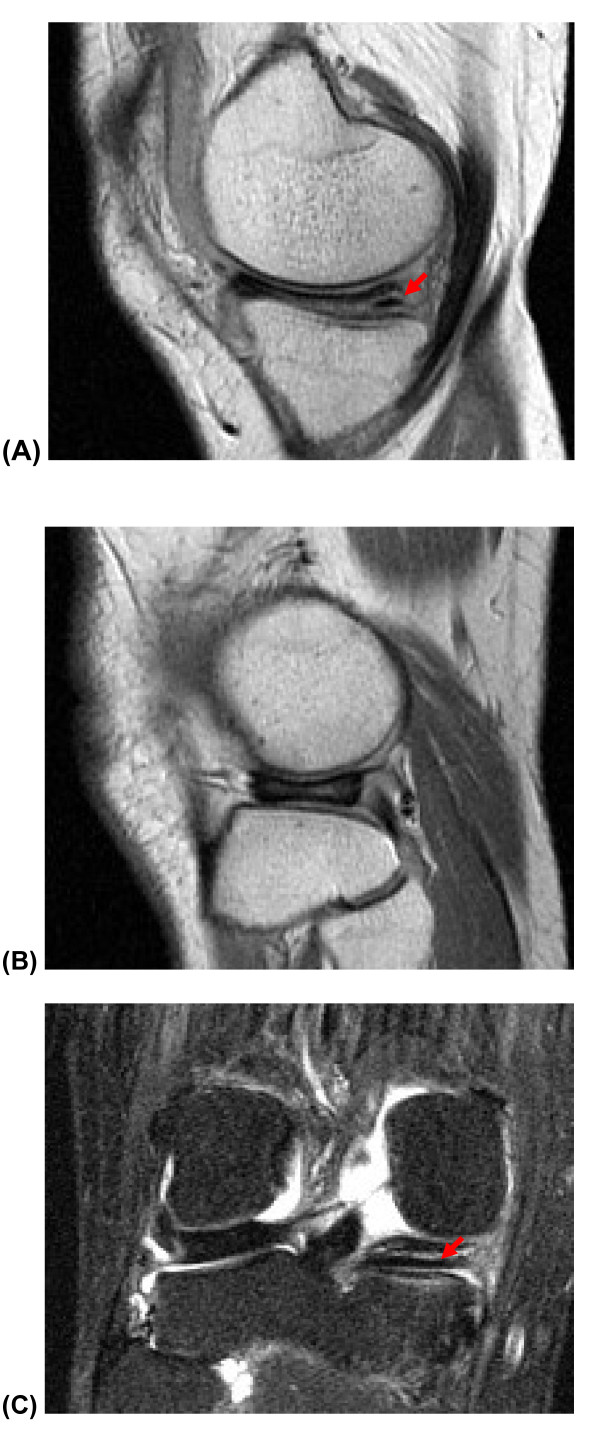
**MRI image of the right knee **(A) T1-weighted sagittal MRI of the right knee showing medial discoid meniscus with a horizontal tear (indicated by red arrow) and (B) showing lateral discoid meniscus; whereas (C) the T2-weighted coronal MRI showing medial discoid meniscus with tear (indicated by red arrow) and lateral discoid meniscus with no tear.

A 44-year-old female patient complained of pain on her right knee after fast walking for two months. On physical examination, the range of motion was limited due to the pain for flexion more than 90°. There was no effusion. The patient had medial joint line tenderness, and a McMurray test elicited pain on the medial joint line. Radiographs of the right knee showed lateral joint space widening, high fibular head, and also increased concavity and subchondral sclerosis of the medial tibial plateau (Fig 1). MRI of the right knee showed a discoid medial meniscus with a horizontal cleavage tear (Fig 2A and 2C) and a discoid lateral meniscus with no tear (Fig 2B and 2C).

**Figure 3:**
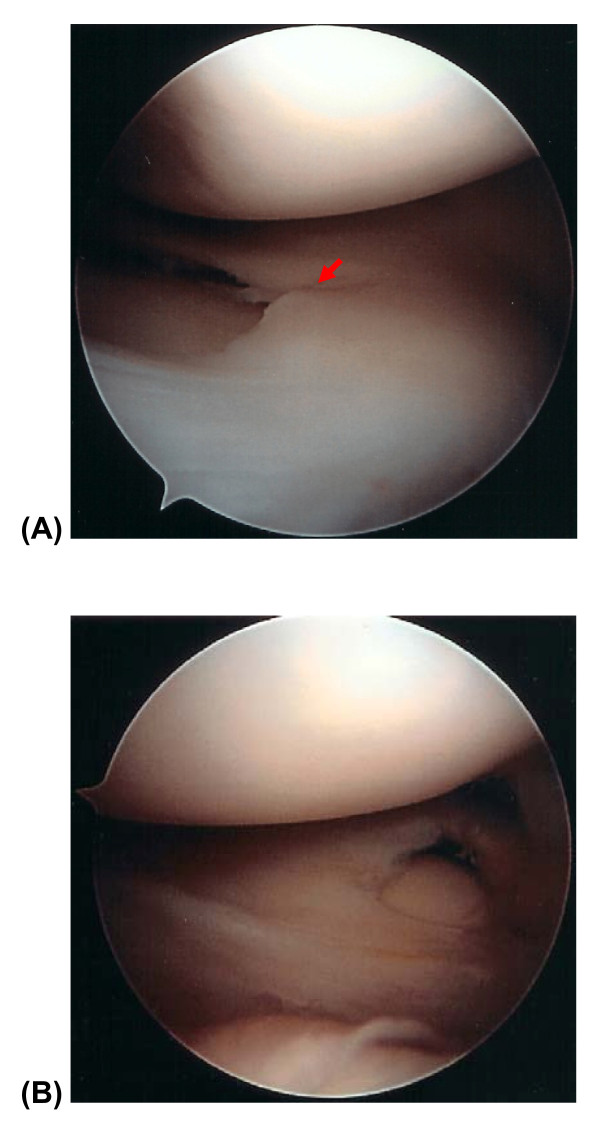
**Arthroscopic view of the right knee**. Arthroscopic examination of the right knee showed (A) an incomplete medial discoid meniscus with horizontal cleavage tear (indicated by red arrow), and (B) a complete lateral discoid meniscus without tear.

Arthroscopic examination of the right knee confirmed the presence of discoid medial and lateral menisci. The medial meniscus was incomplete discoid conformation and had a horizontal tear (Fig 3A), whereas the lateral meniscus was completely discoid and had no tear (Fig 3B). The discoid medial meniscus had an anomalous insertion to the anterior cruciate ligament (ACL). We found also a pathologic medial patellar plica with fibrotic, thickening, and tear. There was cartilage fasciculation on the medial facet of patella. We performed partial meniscectomy of incomplete discoid medial meniscus and resection of pathologic medial patellar plica. We did not perform surgical procedure to the discoid lateral meniscus since the patient had no symptom and no tear. The patient had no limitation of motion or pain 2 years after operation.

**Figure 4:**
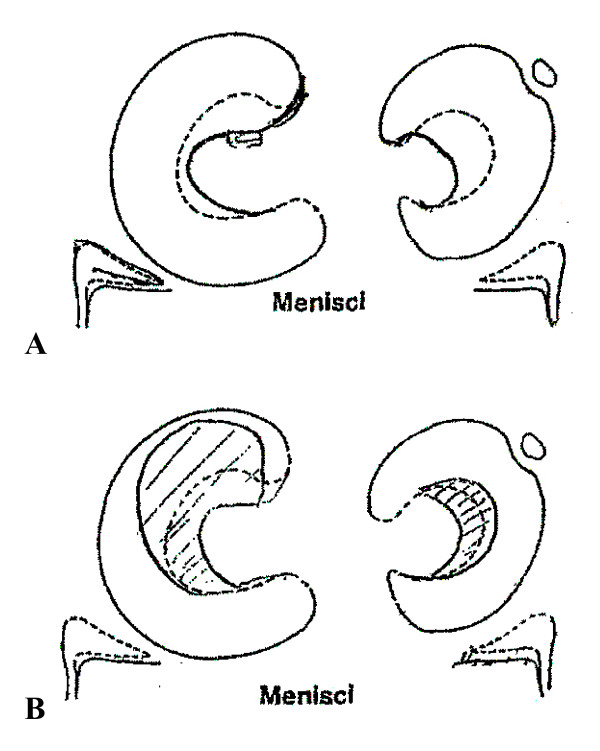
**Original scan operating report done by senior author**. Operating scheme describing incomplete left medial discoid meniscus with flap tear and incomplete left lateral discoid meniscus with horizontal tear. (A) Before meniscectomy. (B) After subtotal meniscectomy of the discoid medial meniscus and reshaping of the discoid lateral meniscus.

From the medical history, it was revealed that seven years before, when she was 37 years old, she complained about pain in her left knee for three years. The pain had become worse during walking. The patient was referred to our hospital under the diagnosis of medial meniscus tear. Physical examination of the left knee showed swelling and medial joint line tenderness. A McMurray test revealed pain with external rotation. Radiographs showed widening of lateral joint space, and a high fibular head, and increased concavity of medial tibial plateau (Fig 1). Arthroscopic examination of the left knee confirmed the presence of incomplete discoid medial meniscus with a flap and horizontal tear, and incomplete discoid lateral meniscus. We performed a subtotal meniscectomy on the medial meniscus and reshaping of the lateral meniscus. Unfortunately, the patient and our hospital did not keep her MRI and arthroscopic picture, so we were unable to present those here. However, we could present the original scan of senior author hand writing operation report describing the operative finding of incomplete discoid medial meniscus with flap and horizontal tear and incomplete discoid lateral meniscus (Fig 4).

On the last follow-up, seven years after the first operation and six months after the second operation, patient had no complaint and satisfied with the result. The radiological examination showed that left knee has apparent varus deformity. It could be due to the prior surgery.

## Discussion

Since Young reported a lateral discoid meniscus, many cases have been found. Discoid lateral meniscus is more common among Asians than that among Caucasians [15-17]. The incidence rate of discoid medial menisci reported was from 0.03% to 0.3% [6,11]. By using MRI, it is now possible to diagnose meniscus condition noninvasively [18]. Blacksin et al. [9] described the first case of bilateral discoid medial menisci diagnosed using MRI. Yanez-Acevedo reported a case of bilateral discoid lateral menisci and unilateral discoid medial menisci, thus three discoid menisci in an 11-year-old girl [19].

Dickason has reported the large series of 10 medial discoid menisci in which there was one 22-year-old man with bilateral massive discoid menisci [6]. The incidence of discoid menisci is difficult to be estimated since the rate of asymptomatic patients is high. Yaniv and Blumberg cited that the incidence of discoid meniscus ranges from 0.4% to 17% for the lateral and 0.06% to 0.3% for the medial side.

Lateral discoid menisci with symptomatic tears are well-known lesions that are usually thought to affect mostly children and adolescent [7,20]. It is also not common for a discoid meniscus to become symptomatic and present with a tear in adulthood [6,7]. However, in the series of Dickason et al., 62% of the patients were older than 18 years of age [6]. Akgun et al. reported bilateral discoid medial menisci in an adult patient [7].

Some papers reported bone changes with discoid medial meniscus [8,21]. Atay et al. [8], reported the increased concavity of the medial tibial plateau of their bilateral discoid menisci case, and decreased signal intensity of the subchondral medial tibial epiphysis consistent with reactive sclerosis. Weiner et al. [21], reported depression of the medial tibial plateau followed by complete reformation of the depressed medial tibial plateau after meniscectomy. In our case, we found bilateral increased concavity of the medial tibial plateau, more clearly in the right side, and sclerotic of the right medial tibial plateau on anteroposterior and lateral radiological examination.

Patel believes that the discoid meniscus should be preserved if "severe symptoms are not present" [22]. The technique used in the management included a careful resection of the menisci back to firm, longitudinal fibers [5]. Senior author has described arthroscopic one-piece excision technique for the treatment of symptomatic lateral discoid meniscus [23].

The result depends on the amount of retained meniscal tissue, the associated lesions, the activity level of the individual, and the length of the follow-up [13]. In general, the results of meniscectomy for discoid meniscus are good. Our patient also had no complain and she has satisfactory with the result of both of her surgeries. However a longer follow-up period is still needed since discoid meniscus has been reported as one of the risk factors for articular cartilage lesions [24,25]. The duration of symptoms and meniscal shape showed significant relation with articular cartilage lesion [24].

## Competing interests

The authors declare that they have no competing interests.

## Consent

Written informed consent was obtained from the patient for publication this case report and the images.

## Authors' contributions

All authors co-wrote the paper and discussed the results for the manuscript preparation. All authors have read and approved the final manuscript.
